# HLA genotyping in the international Type 1 Diabetes Genetics Consortium

**DOI:** 10.1177/1740774510373494

**Published:** 2010-08

**Authors:** Josyf C Mychaleckyj, Janelle A Noble, Priscilla V Moonsamy, Joyce A Carlson, Michael D Varney, Jeff Post, Wolfgang Helmberg, June J Pierce, Persia Bonella, Anna Lisa Fear, Eva Lavant, Anthony Louey, Sean Boyle, Julie A Lane, Paul Sali, Samuel Kim, Rebecca Rappner, Dustin T Williams, Letitia H Perdue, David M Reboussin, Brian D Tait, Beena Akolkar, Joan E Hilner, Michael W Steffes, Henry A Erlich, the T1DGC

**Affiliations:** ^a^Center for Public Health Genomics, University of Virginia, Charlottesville, VA, USA, ^b^Children’s Hospital Oakland Research Institute, Oakland, CA, USA, ^c^Roche Molecular Systems, Inc., Pleasanton, CA, USA, ^d^Clinical Chemistry, University Hospital MAS, Malmö, Sweden, ^e^Victorian Transplantation and Immunogenetics Service (VTIS), Australian Red Cross Blood Services, Melbourne, Victoria, Australia, ^f^Department for Blood Group Serology and Transfusion Medicine, Medical University, Graz, Austria, ^g^Division of Public Health Sciences, Wake Forest University Health Sciences, Winston-Salem, NC, USA, ^h^Division of Diabetes, Endocrinology and Metabolic Diseases, National Institute of Diabetes and Digestive and Kidney Diseases, National Institutes of Health, Bethesda, MD, USA, ^i^Department of Biostatistics, School of Public Health, University of Alabama at Birmingham, AL, USA, ^j^Department of Laboratory Medicine and Pathology, University of Minnesota Medical School, Minneapolis, MN, USA

## Abstract

***Background*** Although human leukocyte antigen (HLA) *DQ* and *DR* loci appear to confer the strongest genetic risk for type 1 diabetes, more detailed information is required for other loci within the HLA region to understand causality and stratify additional risk factors. The Type 1 Diabetes Genetics Consortium (T1DGC) study design included high-resolution genotyping of HLA-*A*, *B*, *C*, *DRB1*, *DQ*, and *DP* loci in all affected sibling pair and trio families, and cases and controls, recruited from four networks worldwide, for analysis with clinical phenotypes and immunological markers.

***Purpose*** In this article, we present the operational strategy of training, classification, reporting, and quality control of HLA genotyping in four laboratories on three continents over nearly 5 years.

***Methods*** Methods to standardize HLA genotyping at eight loci included: central training and initial certification testing; the use of uniform reagents, protocols, instrumentation, and software versions; an automated data transfer; and the use of standardized nomenclature and allele databases. We implemented a rigorous and consistent quality control process, reinforced by repeated workshops, yearly meetings, and telephone conferences.

***Results*** A total of 15,246 samples have been HLA genotyped at eight loci to four-digit resolution; an additional 6797 samples have been HLA genotyped at two loci. The genotyping repeat rate decreased significantly over time, with an estimated unresolved Mendelian inconsistency rate of 0.21%. Annual quality control exercises tested 2192 genotypes (4384 alleles) and achieved 99.82% intra-laboratory and 99.68% inter-laboratory concordances.

***Limitations*** The chosen genotyping platform was unable to distinguish many allele combinations, which would require further multiple stepwise testing to resolve. For these combinations, a standard allele assignment was agreed upon, allowing further analysis if required.

***Conclusions*** High-resolution HLA genotyping can be performed in multiple laboratories using standard equipment, reagents, protocols, software, and communication to produce consistent and reproducible data with minimal systematic error. Many of the strategies used in this study are generally applicable to other large multi-center studies.

## Introduction

Human leukocyte antigen (HLA) genes encode proteins that present antigenic peptide fragments to T-cell receptors and are obvious candidates in the pathogenesis of autoimmune diseases such as type 1 diabetes. Variation in the HLA genes on chromosome 6p21.3 contributes approximately 50% of total genetic susceptibility to type 1 diabetes [1] and the region is maximally linked with the log odds ratio (LOD) score of approximately 116 [2,3]. Although the class II *DQ* and *DR* regions are associated with type 1 diabetes, *DP* and class I *A, B* genes also contribute [4–8]. With 2991 variants presently known (European Molecular Biology Laboratory-European Bioinformatics Institute ImMunoGeneTics/HLA database version 2.20, January 2008, http://www.ebi.ac.uk/imgt/hla/stats.html), alleles of the highly polymorphic HLA genes differ significantly in frequency among ethnic populations and in their association with disease. It remains unclear whether the HLA molecules are causal, and if so, to what extent, or if they are marker linked with the true causal genes. Thus, the Type 1 Diabetes Genetics Consortium (T1DGC) was organized to conduct a large-scale multinational project based on families with affected sibling pairs (ASP) and used comprehensive class I and II genotyping to provide extended haplotypic information that is essential to dissect the relative contributions of specific loci to type 1 diabetes susceptibility, and as a stratifying factor when studying the effects of new candidate genes.

## Materials and methods

### Study populations

**Figure 1:**
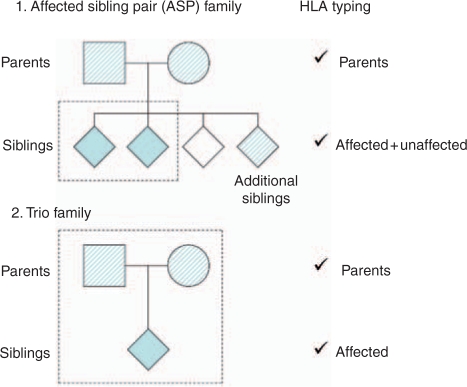
Pedigree structures for families recruited into the T1DGC. Dark fill represents a family member with type 1 diabetes; no fill, unaffected; and crosshatch may be either. The dotted line indicates the minimum inclusion criteria for family recruitment into the T1DGC collection. The maximal included pedigree structure includes 5 affected and 2 unaffected siblings in affected sibling pair families; no additional siblings were collected in trio families. All recruited family members were typed for all HLA loci.

For details on recruitment, participation, and initial sample handling, see the previous articles in this supplement [9,10]. Figure 1 shows the criteria of pedigree structure for inclusion of families and Table 1 the number of samples received at the T1DGC HLA laboratories. All participants gave informed consent in their own language, with specific assent procedures for children, and oversight from at least one ethical review board in each participating country, in accordance with the Declaration of Helsinki [9,11]. In addition to the newly recruited T1DGC families, DNA samples from existing cohorts of families with type 1 diabetes also were genotyped by the T1DGC HLA laboratories using identical protocols, including: HBDI (Human Biological Data Interchange, Philadelphia, PA, USA); BDA (British Diabetes Association Warren 1, Cambridge, UK); Joslin (Joslin Diabetes Center, Boston, MA, USA); Danish (Steno Diabetes Center, Gentofte, Denmark); Sardinian (University of Sassari, Sassari, Italy); UK GRID (United Kingdom Genetic Resource Investigating Diabetes, Cambridge, UK); and B58C (British 1958 Birth Cohort from the National Child Development Study, London, UK).

**Table 1:** Samples received at HLA genotyping laboratories for new T1DGC and existing cohorts, T1DGC, July 4, 2009

Network or cohort	Source network	T1DGC laboratory^a^	Samples received (*N*)^b^
T1DGC affected sibling pair cohorts			
Asia-Pacific	AP	AP	1419
European	EU	EU	5046
North American	NA	NA I + NA II	5180
United Kingdom	UK	EU	1017
Subtotal	**–**	**–**	12,662
T1DGC trio cohorts			
Asia-Pacific	AP	AP	725
European	EU	EU	42
North American	NA	NA I + NA II	564
Subtotal	**–**	**–**	1331
T1DGC case cohorts			
Asia-Pacific	AP	AP	0
European	EU	EU	5
North American	NA	NA I + NA II	169
Subtotal	**–**	**–**	174
T1DGC control cohorts			
Asia-Pacific	AP	AP	0
European	EU	EU	2
North American	NA	NA I + NA II	246
Subtotal	**–**	**–**	248
Existing affected sibling pair cohorts			
Danish	EU	EU	683
Sardinian	UK	EU	348
BDA Warren I^c^	UK	EU^d^	1791
HBDI^e^	NA	NA I + NA II	817
Joslin	NA	NA I + NA II	384
Subtotal	–	–	4023
Existing case cohorts			
UK GRID^f^	UK	AP^d^	570
UK GRID	UK	EU^d^	1518
UK GRID	UK	NA I + NA II^d^	1081
Subtotal	–	–	3169
Existing control cohorts			
B58C^g^	UK	AP^d^	350
B58C	UK	EU^d^	782
B58C	UK	NA I + NA II^d^	722
Subtotal	–	–	1854
Total	**–**	**–**	23,461

a Network HLA laboratories (principal investigators) are: Asia-Pacific (AP): Victorian Transplantation and Immunogenetics Service, Melbourne, Australia (Tait); European (EU): Clinical Chemistry, University Hospital Malmö, Sweden (Carlson); North America (NA): Class I genotyping, NA I, CHORI, Oakland, CA, USA (Noble); Class II genotyping NA II, RMS, Pleasanton, CA, USA (Moonsamy).

b Totals are shown for each network in the T1DGC for affected sibling pair, trio, case and control cohorts, and for each existing, non-T1DGC cohort. The European HLA Laboratory genotyped the United Kingdom Network samples. The North American HLA Laboratory was divided into two physically separate laboratories: NA I and NA II.

c BDA Warren I: British Diabetes Association Warren I.

d Samples typed for HLA–*DPA1, –DPB1*, –*DQA1*, and –*DQB1* loci only.

e HBDI: Human Biological Data Interchange.

f UK GRID: United Kingdom Genetic Resource Investigating Diabetes.

g B58C: British 1958 Birth Cohort from the National Child Development Study.

### Laboratory equipment

Uniform laboratory equipment was provided for all sites. Equipment included: the Symbol D4000i barcode reader (Symbol Technologies Inc., Holtsville, New York, USA); Applied Biosystems 9600 thermal cyclers for polymerase chain reaction (PCR; a single exception being the use of the pre-existing Applied Biosystems 9700 thermal cyclers at the European laboratory); a BeeBlot hybridization incubator (Bee Robotics Ltd, Caernarfon, Gwynedd, Wales, UK) with its operative program; and an Epson 1670 flatbed scanner (Seiko Epson Corp., Nagano, Japan).

### Reagents

All laboratories used the same batches of PCR master-mix, immobilized probe linear arrays (also known as ‘line strips’), and development reagents, provided by Roche Molecular Systems (RMS), Pleasanton, CA, USA [12] with careful documentation of batch numbers and with inter-batch comparisons. The linear arrays used in the T1DGC are currently not commercially available.

### Software

A T1DGC custom-developed HLA Laboratory System was used throughout for HLA sample and assay tracking. The web-enabled relational database system automatically creates 96-well plate grids for each sample shipment (allowing laboratories to create new grids for repeats); sample interface files for StripScan; and supports upload and quality control (QC) checking of HLA genotype data files exported from Sequence Complication and Rearrangement (SCORE) in Extensible Markup Language (XML) format, delivered through web page upload to the T1DGC central database at the Coordinating Center.

#### StripScan

During the project, RMS developed and made available the StripScan program for linear array HLA genotyping analysis. It imports signal intensity for each probe on an array from the flatbed scanner, detects each probe as positive or negative (1, 0), and assigns a confidence score to each probe call. Using a distance algorithm, it determines the most likely genotypes from the strip pattern and provides a confidence score for each genotype, indicating proximity to the observed probe pattern. This result is reviewed, accepted, or altered by the laboratory personnel, and a report file containing the signal intensities and probe calls for each strip is saved and imported into SCORE for final processing and transmission of the results.

#### SCORE

SCORE is a PC-based software program used by many HLA genotyping laboratories and studies [13]. SCORE allows for the review of probe binding patterns and final selection of the accepted HLA genotype calls. This review is completed prior to the export of plate grid genotype data in XML format for transmission and upload to the Coordinating Center website. The XML file contains the genotype call (two alleles at four-digit resolution) at eight loci for each sample on the plate, probe intensities, and probe detection patterns.

At the Coordinating Center, QC checks of genotype data were performed using SAS (SAS Institute, Cary, NC, USA). The program PEDCHECK (HLA modified version) [14] was used to identify Mendelian inheritance errors (MIEs) for single HLA gene alleles. Extended *A-B-C-DRB1-DQ-DP* haplotypes were reconstructed using Merlin software [15] and checked for obligate recombination.

### Training

After the selection of equipment and development of protocols, nomenclature, databases, and software by the T1DGC-nominated reference laboratories (RMS; Children’s Hospital Oakland Research Institute, CHORI), a one-week training course for the laboratory principal investigators and technicians from T1DGC networks (Asia-Pacific, European, and North America) was conducted. Then, an initial certification testing (ICT) exercise was performed at each local network laboratory using a panel of 20 unrelated, mixed-ethnic group, DNA samples provided by The Fred Hutchinson Cancer Research Center (Hansen laboratory, Seattle, WA, USA). This panel had been previously HLA genotyped to equivalent high resolution by non-T1DGC HLA laboratories and contained samples with both common and rare alleles. The local network laboratories were not informed of the ethnicity of the participants.

The initial standard for certification was 0% discordance in allele comparisons with existing genotypes, as judged by the HLA Laboratory QC Committee. No single laboratory performed with 100% concordance for alleles and as issues concerning data reporting standards, allele nomenclature, and ambiguities became evident, a ‘retraining workshop’ was organized. Certification criteria were adjusted to more realistic goals, including data transmissions that satisfied T1DGC genotype calling standards; 0% discordance rate at the four-digit level for disease-critical *DQ, DR* locus alleles, with 0% error in two-digit allele group calls and 98% agreement at four-digit allele resolution for the other five loci. In a second exercise, each HLA Laboratory typed an identical standard panel of 20 cell line samples selected from the previously sequence typed DNA samples provided by the Centers for Disease Control and Prevention (CDC, Mueller laboratory, Atlanta, GA, USA). The laboratories were notified of the ethnicity of the participants (three Hispanic, two African American, five Asian, and 10 Caucasian).

### DNA sample handling and tracking

The DNA from the T1DGC families was shipped in boxes of 92 screw-capped tubes with bar-coded labels (5 µg of DNA at 20 ng/µL; total volume 250 µL) by the DNA repositories. Family member samples were grouped on plates whenever possible. Each plate also contained four water (blank) controls at constant asymmetrical positions, as a way to determine plate orientation and potential contamination during subsequent processes. The barcodes for the sample indicate the network of origin, a family code, and a suffix to indicate father (01), mother (02), proband (03), or sibling (04–09). Information on ethnicity also was transmitted. The laboratories acknowledged the receipt of each shipment by entry of the shipping forms into the T1DGC HLA Laboratory System, including scanning of the barcode for each sample. The samples were spun briefly and pipetted into a 96-well plate according to a plate grid automatically generated for the laboratory by the HLA Laboratory System. Each of the six RMS linear arrays (*A*, *B*, *C*, *DQ*, *DRB1*, and *DP*) requires a separate 96-well plate.

### HLA genotyping

**Figure 2:**
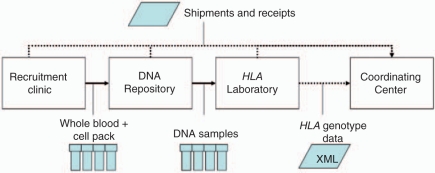
Simplified process diagram showing HLA genotyping-related specimen and data flow within a T1DGC network (Asia-Pacific, Europe, North America, and the United Kingdom).

PCR co-amplification of exons 2 and 3 for HLA class I assays and amplification of exon 2 for HLA class II was performed using 60 ng template genomic DNA, biotinylated primers, and reagents from RMS in a 60 µL reaction mix, and a standardized PCR protocol (denaturation at 95°C for 15 s, annealing at 60°C for 45 s, extension at 72°C for 15 s for 35 cycles with an additional 72°C 5 min hold and a 15°C hold on an Applied Biosystems 9600 thermal cycler; as a single exception, the European laboratory used 58°C as the annealing temperature for *DQ* amplification on the Applied Biosystems 9700 thermal cycler). Using sequence-specific immobilized oligonucleotide probe (SSOP) linear array technology [12], each biotinylated PCR product was hybridized to the relevant series of unlabeled oligonucleotide probes immobilized on nylon-backed membrane arrays, corresponding to DNA sequence motifs in a given HLA gene locus, in linear batches of 48 wells. A full HLA genotype profile for a single sample required one linear array each for the *A* (57 probes), *B* (81 probes), *C* (36 probes), *DQ* (15 *DQA1* and 37 *DQB1* probes), and *DP* (21 *DPA1* and 48 *DPB1* probes) loci. A low-resolution *DRB1* array (8 probes) identified the major WLF, WPR, YSTS, VH, YSTG, GYK, KDF, and EV codon 10–14 motifs as well as two probes each for the *CTLA4* T17A (rs231775) single nucleotide polymorphisms (SNP) and the *INS*-23 *HphI* (rs689) SNP. For rare homozygotes of the *DRB1* *0901 or *1001 alleles, no further *DRB1* genotyping was required. For other classes, a second high resolution *DRB1* linear array (31 probes) was used following an allele-specific PCR for each allelic class identified by the low-resolution array. The BeeBlot automated hybridization instrument performed a temperature-controlled program of hybridization and aqueous washing. After development with streptavidin, horse radish peroxidase, and substrates, the blue signals on the array were scanned on a flatbed scanner and the resulting digital image was processed in StripScan software. Results from StripScan were transmitted to the SCORE program for a final genotype review, assignment, and approval. Selection from among all the possible suggested genotypes was based on experience and consistency within families and haplotype structure. After the approval of genotypes at all loci for the 96 samples, the genotypes and probe call intensities were uploaded to the Coordinating Center in XML format using the HLA Laboratory System (Figure 2). Throughout production, only in North America (NA), the NA I Laboratory (CHORI) genotyped all class I loci and NA II (RMS) genotyped all class II loci.

### HLA plate grid and genotype data quality checks

Upon receipt at the Coordinating Center, the XML data file passed through a software pipeline of data quality and consistency checks to ensure: (1) process consistency (*i.e.*, concordance with expected plate grid, position, and sample); (2) consistency with T1DGC HLA nomenclature and allele calling standards; and (3) genetic inheritance consistency, using PEDCHECK and Merlin. Data checks were performed in real time to provide rapid feedback and implementation of corrective measures for potential errors.

### Quality control

Four samples from a previously genotyped family were randomly selected by the Coordinating Center, re-coded, and included in each plate for continuous QC. HLA laboratories were blinded to these samples and their identifiers. In addition, all laboratories participated in an annual QC test in which each performed single-blind re-genotyping of an identical panel of 92 samples. These samples were selected by the Coordinating Center from approximately 24 ASP families (eight from each network) and previously typed by one of the laboratories in normal production, but not previously used as QC samples. Thus, approximately one-third of the genotype results provided a test of intra-laboratory reproducibility and all 92 samples measured inter-laboratory concordance.

## Results

### Initial certification testing

Each laboratory (including both NA class I and II laboratories in North America) typed all loci in initial certification tests. The results obtained for the second CDC panel were compared to the CDC-known reference genotypes (Table 2). Some discrepancies between the T1DGC consensus genotype and the CDC genotype were due to allele ambiguities, *i.e.,* multiple alleles consistent with the genotype probe pattern with no distinguishing sequence motifs on the T1DGC linear arrays (data not shown in CDC comparison). The consensus allele calls of all T1DGC laboratories were 99.1% concordant with CDC calls, with discrepancies in 3/320 allele calls, in *B* and *C* class I loci. At two-digit resolution, the three discrepant alleles were concordant with CDC. Within T1DGC comparisons, 13 total discordant alleles were seen (99.0% overall concordance), but 7/13 were due to the differences in ambiguous allele reporting (all *DQA1*). These differences highlighted the necessity of standards for handling allele ambiguity in the T1DGC to ensure consistency of allele calls between laboratories. After adjusting for ambiguity, the overall concordance was 99.5% and all remaining allele discrepancies were in class I loci and consistent at two-digit resolution. The other result of the ICT exercises was the development of T1DGC standards for HLA genotype and allele calling to address issues of ambiguity and data completeness (Appendix).

**Table 2:** HLA Laboratory initial certification testing results^a^, by laboratory comparison and locus, T1DGC, July 4, 2009

	*A* (%)	*B* (%)	*C* (%)	*DPA1* (%)	*DPB1* (%)	*DQA1* (%)	*DQB1* (%)	*DRB1* (%)	Total (%)
Comparison									
T1DGC vs CDC^b^	–	1 (97.5)	2 (95.0)	–	–	–	–	–	3 (99.1)
North American I vs T1DGC	–	–	–	–	–	2 (95.0)	–	–	2 (99.4)
North American II vs T1DGC	–	–	–	–	–	–	–	–	–
Asia-Pacific vs T1DGC	–	2 (95.0)	1 (97.5)	–	–	5 (87.5)	–	–	8 (97.5)
European vs T1DGC	–	2 (95.0)	1 (97.5)	–	–	–	–	–	3 (99.1)
Total	–	5 (96.9)	4 (97.5)	–	–	7 (95.6)	–	–	16 (98.7)

a Allelic concordance is shown at the allele level for T1DGC consensus genotypes compared to CDC, and individual T1DGC HLA laboratories compared to the consensus. ‘–’ indicates 100% concordance. Other counts are the numbers of discordant alleles (% concordance). These results are for the second blinded initial certification testing as described in the text. Each laboratory typed 20 unrelated, mixed-ethnicity samples (40 alleles × 8 loci).

b All discrepancies between T1DGC consensus and CDC are due to ambiguities, *i.e.,* identity within the tested exons.

### Continuous quality monitoring

The way in which data were acquired for plate grids, probe intensities, probe binding patterns, and genotypes enabled analysis of consistency in reagent batches, changes in protocol, and data interpretation. Signals for the water controls in each plate revealed general and specific contamination levels or different array washing stringencies in laboratories (data not shown). Intra-laboratory concordance of the repeat genotyping of the internal blinded QC family samples (four from a single family per plate) showed an overall allele concordance of 99.3% (Table 3). The discordant alleles were reasonably evenly distributed across all loci, although *DPA1* had no discrepancies.

**Table 3:** Intra-laboratory allelic percent concordance for blinded continuous quality control testing^a^, by HLA locus, T1DGC, July 4, 2009

Source network	Plates	Quality control samples	*A*	*B*	*C*	*DPA1*	*DPB1*	*DQA1*	*DQB1*	*DRB1*	SNP^b^: *CTLA4*	SNP: *INS-23 HphI*	All loci
Asia-Pacific	22	87	–	–	–	–	–	–	–	–	–	98.9	99.9
European	55	219	–	99.1	–	–	99.5	–	–	98.2	98.6	–	99.5
North American I	60	238	97.9	98.3	99.6	–	–	–	–	–	–	–	98.6
North American II	60	238	–	–	–	–	97.9	99.6	99.2	98.7	99.2	99.2	99.1
United Kingdom	6	24	–	–	–	–	–	–	–	–	95.8	–	99.6
Total	143	568	99.1	98.9	99.8	–	98.9	99.8	99.6	98.8	98.9	99.5	99.3

a Four quality control samples are included on each plate. ‘–’ indicates 100% concordance between original and blinded quality control repeat alleles. Empty cells for North American (NA I and NA II) Laboratories are the HLA class loci that they do not genotype. Identical samples were assayed separately by NA I and NA II and are only counted once in plate and sample totals. The European Laboratory genotyped all United Kingdom Network samples.

b SNP: single nucleotide polymorphisms.

### Annual QC tests

Three annual QC testing exercises were conducted from 2005 to 2007. Table 4 shows intra- and inter-laboratory concordance rates for HLA alleles for all laboratories. Intra-laboratory discrepancies reflect the differences between the annual QC result and the result originally reported by the same laboratory for the same sample. The three network laboratories individually showed ≥99.4% internal concordance each year. An inter-laboratory discordance occurs if the result for one allele differs from those of the other two laboratories. In both intra- and inter-laboratory comparisons, multiple discrepancies within the same locus and laboratory always occurred within a single family, often the result of different interpretations of a single weak probe. In 2006, all laboratories were concordant for HLA*-C* in one family, but all were discordant from the original genotype reported. This discrepancy was also due to an interpretation of a single probe and followed intensive review of the particular genotype. This discrepancy is therefore reported as intra- and not inter-laboratory discordance. The three-way inter-laboratory concordance rate per total number of alleles reported was 99.7% for 2005–2007 combined.

**Table 4:** Intra- and inter-laboratory results of annual quality control testing of HLA laboratories measured by concordance of alleles in genotypes compared (*n*/total and % of total), T1DGC, July 4, 2009

Annual quality control	2005	2006	2007
Quality control sample network source			
Total samples	92	90^b^	92
AP/EU/NA^a^	32/32/28	32/32/26	30/30/32
Total families	25	23	25
AP/EU/NA	9/9/7	8/8/8	8/8/9
Total alleles	1472	1440	1472
AP/EU/NA	512/512/448	512/512/416	480/480/512
Intra-laboratory concordance analysis			
Alleles *N*/total (% concordance)			
Asia-Pacific	30/32 (99.6)	30/32 (99.6)	29/32 (99.4)
European	32/32 (100)	32/32 (100)	32/32 (100)
North American^c^	28/28 (100)	25/26 (100)	28/28 (100)
*Lab*: HLA locus Discrepancies (*N*)	*EU*: *B* (1)	*AP*: *C* (2)	*AP*: *A* (3)^d^
	*NA*: *DPA1* (1)	*NA*: *C* (1)	
Inter-laboratory concordance analysis			
Three-way concordant alleles *N*/Total (% concordance)	1470/1472 (99.9)	1431/1440 (99.4)	1469/1472 (99.8)
*Lab*: Locus discrepancies from consensus (*N*)	*AP*: *A* (1), *B* (1)	*AP*: *A* (1), *C* (1), *DRB1* (1) *EU*: *C* (1), *DPB1* (3) *NA*: *B* (1), *C* (1)	*AP*: *A* (1), *DPB1* (1) *EU*: *B* (1)

a AP: Asia-Pacific; EU: European; NA: North American.

b Two samples were not included in results due to sample mix-up in quality control plates. ^c^North American results are the combined total for NA I and NA II laboratories.

d Asia-Pacific reported three discordant *A* alleles compared to its own original genotyping. However, all three T1DGC labs showed consensus on the quality control genotyping suggesting the same original allele in all three samples from one Asia-Pacific family was incorrect.

### Genotype QC analysis: Mendelian inheritance checks

In each network, a small number of pedigrees displayed apparent MIEs based on transmission of alleles and haplotypes from the parents to the offspring. MIEs may be due to sample mix-up at any of three handling stages (incorrect registration of parenthood, incorrect genotyping, or sample contamination); discrepancies between self-reported and biological familial relationships; or true *de novo* mutations. Attempts at resolution involved multiple steps of review and repeat genotyping. For pedigrees with inconsistencies at two or less HLA loci, genotyping was reviewed, repeated, or supplemented by DNA sequencing. MIEs at three or more loci were interpreted as most likely due to sample mix-up or inconsistency of biological and self-reported relatedness and were referred to the DNA repository and Coordinating Center for review, and possible provision of new samples and repeat genotyping.

Table 5 shows the cumulative number of MIEs identified by network cohort and the results of follow-up analysis. Overall, 3.7% of families (161/4355 total) contained one or more MIEs, of which 23.0% were most likely due to genotyping errors, 19.9% to sample mix-up, and 51.6% to biological nonrelatedness. Potential explanations among the remaining unresolved nine pedigrees (5.6%) with MIEs may be early sample contamination or *de novo* mutation.

**Table 5:** Total Mendelian inheritance errors (MIEs) within families and most likely cause, by cohort and laboratory^a^, T1DGC, July 4, 2009

	Families typed		Identifiable (most likely) cause (*N* and % of all MIEs)			
Source of T1DGC/existing cohort family	Total (*N*)	Number w/MIE (%)	Genotyping error	Sample mix-up	Cryptic relatedness	Unresolved
T1DGC cohorts						
Asia-Pacific	579	26 (4.5)	12 (46.2)	4 (15.4)	6 (23.1)	4 (15.4)
European	1287	23 (1.8)	3 (13.0)	7 (30.4)	13 (56.5)	0 (0.0)
North American I	1385	32 (2.3)	7 (21.9)	15 (46.9)	10 (31.3)	0 (0.0)
North American II	1385	33 (2.4)	8 (24.2)	15 (45.5)	10 (30.3)	0 (0.0)
United Kingdom	169	3 (1.8)	2 (66.7)	1 (33.3)	0 (0.0)	0 (0.0)
Subtotal	3420	92 (2.7)	32 (34.8)	27 (29.3)	29 (31.5)	4 (4.3)
Existing cohorts						
European	225	24 (10.7)	0 (0.0)	0 (0.0)	24 (100.0)	0 (0.0)
North American I	286	5 (1.7)	2 (40.0)	1 (20.0)	2 (40.0)	0 (0.0)
North American II	286	6 (2.1)	3 (50.0)	1 (16.7)	2 (33.3)	0 (0.0)
United Kingdom	424	37 (8.7)	0 (0.0)	4 (10.8)	28 (75.7)	5 (13.5)
Subtotal	935	69 (7.4)	5 (7.2)	5 (7.2)	54 (78.3)	5 (7.2)
Total	4355	161 (3.7)	37 (23.0)	32 (19.9)	83 (51.6)	9 (5.6)

a Results are reported as number of families with ≥1 MIE (*N*) and percentage of total MIEs (%) and shown for each HLA Laboratory, stratified into T1DGC and existing cohort families. In NA, separate HLA Laboratories genotype HLA class I and class II linear arrays, but results for the NA I + NA II Laboratories are combined and North American families are counted once. Sample mix-up means prior to HLA Laboratory sample handling, *i.e.,* at the recruitment clinic or network DNA Repository. Cryptic relatedness means that there is most likely a discrepancy between self-reported and biological relatedness within the genotyped family. The European Laboratory assayed the United Kingdom T1DGC and existing cohort family samples.

### Assay repeats

**Figure 3:**
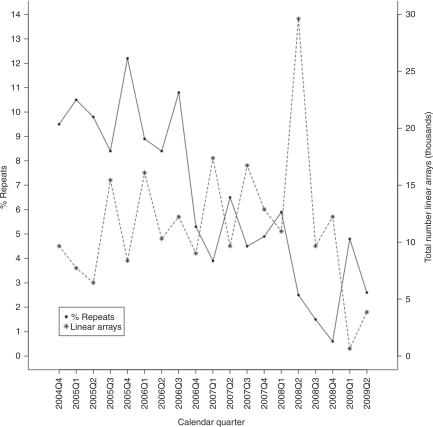
Repeated linear array assays for all T1DGC laboratories as a function of calendar time period.

The number of assay repeats performed at each laboratory was also monitored continuously (Figure 3). The significant trend of decreasing rates of assay repeats (–0.59% per quarter, *p* > 0.0001) reflects a learning curve in assay interpretation, consistency of reagent batches, and resolution of some issues concerning the identities and quality of DNA delivered to a laboratory. Linear array-specific repeat data (Table 6) also reflect the complexity of interpreting probe patterns for different HLA loci and the ethnic complexity within the populations studied. The current cumulative repeat rate is 5.9%.

**Table 6:** Percent repeated assays, by HLA linear array type and laboratory^a^, T1DGC, July 4, 2009

Source	Samples	*A*	*B*	*C*	*DQ*	*DP*	*DRB1* (low)	*DRB1* (high)	Total (%)
Asia-Pacific	3064	10.9	7.6	8.8	9.9	6.5	9.7	7.2	8.6
European	5778	5.5	2.3	5.3	4.9	2.1	5.9	4.1	4.3
North American (class I)	9163	9.7	7.6	9.7	–	–	–	–	9.0
North American (class II)	9163	–	–	–	6.8	4.5	3.9	5.6	5.2
United Kingdom	4674	3.6	6.2	3.4	11.0	8.8	1.6	1.7	5.2
Total	22,679	5.3	3.8	5.0	5.3	3.3	4.0	3.7	5.9

a North American sample repeats are stratified into class I and class II, corresponding to North American I and North American II laboratories; empty cells are HLA class loci that they did not genotype. The total sample count includes the North American samples once. The European Laboratory assayed the United Kingdom Network samples.

## Discussion

We have implemented processes and systems to support high volume, high resolution HLA genotyping for an international consortium across four geographically separate HLA laboratories located on three continents. We standardized operations as much as possible to eliminate extraneous sources of variability in assay platform, reagents, assay conditions, software versions, software interfaces, and data reporting. When required, the assay reagent supplier (RMS) rapidly shipped new lots of reagents to T1DGC HLA laboratories and has provided comprehensive support to the T1DGC to ensure the highest quality genotyping results. Over time, laboratories became more experienced with the assay, evidenced by the decrease in repeat assays.

We found that initial laboratory training was important, but unforeseen issues arose with assay protocol and genotype reporting, and T1DGC performed the second ICT exercise. This second exercise reinforced the value of the initial blinded panel testing to establish laboratory expertise and familiarity with the study protocols and to test processes.

Annual laboratory testing through a periodic blinded QC testing process, after the completion of the ICT panels at the beginning of genotyping operations, enabled us to compare inter-laboratory variation as well as variation within a laboratory over time. Thus, we could monitor long-term quality and estimate cumulative error rates. The blinded retesting of laboratories is similar to a periodic re-accreditation process for laboratory certification, such as used by the American Society of Histocompatibility and Immunogenetics (http://www.ashi-hla.org) and University of California at Los Angeles (UCLA) Exchange programs (http://www.hla.ucla.edu/cellDna.htm). Comparison of annual QC results from 2005 to 2007 showed consistently high rates of intra- and inter-laboratory concordance with no evidence of a significant trend. Out of the total eight intra-laboratory discordances in three years, only one was for a class II locus. For inter-laboratory discordances, only 5/14 were for class II. The greater discordance rate for class I (*A*, *B*, and *C*) loci was not surprising, given the greater locus allelic diversity and larger numbers of probes on the arrays. Further analysis of the familial distribution of discordant alleles also revealed that the error rates depend critically on the family structure, which influences the number of copies of an allele in a family. Error rates were higher for single copy alleles not transmitted by parents to offspring or for families without recruited parents.

Monthly conference calls included the assay and reagent supplier; their inclusion permitted coordination of shipments and direct communication of assay performance. An example of an assay adjustment was a slight change in the hybridization temperature to resolve selective allele dropout and faint array probes. Strong cooperation between the laboratories meant that we were able to compare experiments in the different laboratory environments to verify the conditions under which allele problems occurred and yielded much more standardized calls of alleles.

We invested significant time and resources to develop software and standardized computer systems, which was a considerable upfront cost to the study. This investment brought benefits, including: consistent data reporting; streamlined laboratory data entry, and assay setup; reduced effort by laboratory staff and analysts to manually compile data and generate reports; and accurate final data sets for analysis. Many of these benefits are easily overlooked once these systems are operational.

Distributed HLA genotyping across laboratories in multi-center studies permits assessment of inter-laboratory variability of assays compared to a single centralized laboratory and improves geographical proximity to recruiting centers. This structure minimizes transportation and administration costs (especially costs associated with government approval of export of biological specimens and extra coordination of sample shipments) and sample degradation or mishandling. However, some may decide that these advantages are more than outweighed by the disadvantages of additional coordination of study assay quality and organizational complexity, and adopt the alternative model of a single centralized core laboratory instead. Many of the QC issues raised are still pertinent and the solutions adopted in the T1DGC would be appropriate and easier to implement and monitor in a single laboratory organization.

## Limitations

The HLA genotyping process used a single PCR reaction to generate co-dominant sequence templates at exons 2 and 3 for each class I locus, and exon 2 for each class II locus. A restricted number of probes interrogate most nucleotide polymorphisms; therefore, allelic variation outside of the genotyped exons, or variation with no hybridizing probe within the analyzed exons is not detected. Uncertainty concerning which allele contains a particular polymorphism may also exist. The process produces a probe pattern that is compatible with more than one allele combination, *i.e.,* allele calling may be ambiguous. To obtain a completely unambiguous genotype would require multiple PCR reactions and linear arrays with many more probes per locus or the use of alternative technology such as resequencing. These were either not available or not feasible, because of cost at the commencement of the study. T1DGC laboratories elected to select the most likely genotype call, using information from family allele transmissions and observed allele frequencies; a standard allele designation for ambiguous groups of alleles was used. The likelihood of an incorrect allele assignment will be the subject of review at the completion of the study.

## Conclusions and recommendations

The lessons learned by the international T1DGC in setting up distributed laboratory HLA genotyping, and the general procedures implemented to maintain the highest possible assay quality and reproducibility, are relevant to any national or international multi-center study confronted with the challenge of managing assay quality across separate laboratories. The distribution of HLA genotyping among several laboratories has increased some administrative tasks, but has reduced shipping costs, reduced sample damage during shipping, and has facilitated governmental approval for export of biological samples as compared to a single central laboratory. It exposed weaknesses in the processes for reporting assay results, enabled assessment of inter-laboratory variability of assays, and improved understanding of technical problems.

The complexity and variability of the HLA genome region has limited the number of samples or resolution of genotyping in previous reports of HLA association with disease, such that the results obtained with different technologies are not always comparable. The T1DGC implemented processes and systems that supported high resolution, four-digit HLA genotyping at eight loci with a remarkably high level of consistency across four international laboratories on multiple continents. This level of consistency was achieved through the use of uniform reagents, protocols, instrumentation, software, automated data transfer, continuous QC, and communication. The simultaneous genotyping of multiple participant family members enabled accurate haplotype reconstruction and was of great importance for correct genotyping.

In the T1DGC, the Coordinating Center developed a centralized, web-deployed sample shipment and laboratory assay tracking system that the laboratories integrated with their local laboratory management system. Since there are few off-the-shelf software packages that adequately address international sample shipment functions for multi-center studies without requiring the implementation of complex corporate inventory and shipping packages, the T1DGC almost invariably required custom software development. The RMS system used in the T1DGC is a non-commercial expanded version of a commercial product and so differences in experience with the specific HLA assays were expected.

In addition to the large, newly recruited T1DGC cohort, additional genotyping performed by the same method on samples from other existing cohorts will complement available results to create a large homogeneous database for current and future statistical analysis. To date, the consortium has generated HLA genotypes for over 22,000 samples with an overall concordance of >99.3% achieved.

We offer the following recommendations for large studies, recognizing that the implementation will depend on the scope, organization, and goals of the study.

1. Standardize assay platform and protocol for all laboratories. If possible, use identical technology and laboratory instruments (manufacturer and version).
2. Utilize barcode labeled tubes and readers to minimize data entry of identifiers, with barcode reading software to provide checksum-based error checks on scans to reduce data entry errors.
3. Develop common processes and software to manage transmission of sample shipment and assay result data between the laboratories and the Coordinating Center.
4. Automate data transfer between software programs wherever possible. Again, this automation will usually require custom software development to build programmatic interfaces, but will reduce or eliminate the need for data re-entry and data errors.
5. Standardize software, allele calling database, and algorithms used to analyze assay data.
6. Conduct pre-production training. Laboratories often use different assay protocols and/or technology and have less experience with methods in use elsewhere. In HLA genotyping, different methods exist including sequencing and SSOP genotyping.
7. Conduct a blinded pre-production initial certification or proficiency test using common samples with known assay titers or assay results. For HLA genotyping, use a common panel of DNA samples previously genotyped to comparable resolution. Require each laboratory to demonstrate initial proficiency in the assay procedures, before performing production assays on participant samples. Laboratories may need to repeat this exercise with new panels of samples if the first ICT reveals lower than desired concordance of assay results. Develop realistic certification metrics prior to testing.
8. Implement a rigorous and continuous QC program. Over the lifetime of a multi-year project, there can be a longitudinal drift in the assay quality and reproducibility. This drift may be associated with readily identifiable factors such as new technical staff, changes in laboratory environment, changes in reagent batches, or less obvious reasons.
9. Define standards for assay analysis and data reporting to the Coordinating Center. In the case of HLA genotyping, these standards should cover representation of alleles (digit resolution, with or without locus prefix, homozygotes); genotyping platform resolution-dependent allele ambiguities; and strategy for handling new alleles. The standards will eliminate confusion and ensure that the study database maintains consistent power for analysis.
10. Implement a system of QC checks in the Coordinating Center, after laboratories have performed their analysis of raw assay data and transmitted the results to the Coordinating Center. The system should verify that the reported data meets study assay reporting standards, and may repeat QC performed in the laboratories, perhaps with additional automation not possible locally. For genotyping, these checks could include tests of Hardy–Weinberg Equilibrium, Mendelian Inheritance, cryptic sample duplicates (sample mix-ups) or sample familial relationships, sample contamination, and sample biological sex.
11. Plan for ongoing review of the best laboratory practices and assay performance issues through regular meetings and conference calls.

The management of laboratory assay quality is an important consideration for any large study, but is especially challenging in a study with multiple laboratories. Implementation of standard processes and QC procedures can significantly improve assay and data quality, but can be a complex undertaking, requiring compromise, flexibility, and, above all, regular communication.

## Appendix: T1DGC HLA genotype and allele calling standards

1. Genotype calls must be reported with full four-digit resolution. Non-coding change variants should not be reported (*i.e.,* no five or six-digit resolution alleles).
2. Two alleles for each locus must be explicitly reported to the Coordinating Center, including homozygotes.
3. Alleles that are ambiguous using the approved assays will be reported as the lowest numerical allele in the ambiguity group.^1^
4. Newly discovered alleles will be assigned a temporary designation based on the closest existing allele (by sequence) prior to sequencing, registration, and receipt of a new official allele designation from the International Immunogenetics HLA Database Project IMGT/HLA (http://www.ebi.ac.uk/imgt/hla). Sequencing and registration are not obligatory within the T1DGC, although laboratories may collaborate with the recruiting investigator to characterize new alleles.
5. The same version of the HLA allele database [16] must be used in the HLA genotype calling software in all laboratories (StripScan and SCORE), with synchronized updates across laboratories.
6. All probes must be called present or absent consistent with the final genotype, and not left indeterminate (*i.e.,* ‘weak’ setting). The probe binding pattern (string of all probe detection calls for a linear array) and probe intensities must be transmitted to the Coordinating Center with the genotypes.

## Note

1. The National Marrow Donor Program system [17] to identify intermediate resolution ambiguous allele groups (http://bioinformatics.nmdp.org/HLA/Allele_Codes/Allele_Code_Lists/index.html) was not utilized in this study due to incompatibility with the allele databases and genotyping kits in the software version used and difficulties in validating allele group membership.
